# A curative effect evaluation of intensity-modulated radiation therapy combined with periorbital triamcinolone acetonide injection in treating thyroid eye disease patients with active extraocular muscle but low CAS

**DOI:** 10.1038/s41598-025-88142-w

**Published:** 2025-01-25

**Authors:** Hao Zhang, Weimin He

**Affiliations:** Department of Ophthalmology, West China Hospital of Sichuan University, No.37, Guoxue Xiang, Chengdu, 610041 Sichuan China

**Keywords:** Thyroid eye disease, Intensity-modulated radiation therapy, Clinical activity score, Magnetic resonance imaging, Outcomes research, Eye abnormalities

## Abstract

The purpose of this study is to evaluate the effectiveness of intensity-modulated radiation therapy (IMRT) combined with periorbital triamcinolone acetonide injection in treating thyroid eye disease (TED) patients with active extraocular muscle but low CAS. The retrospective observational study was conducted. A total of 156 eligible patients were selected from the TED patient database of the Ophthalmology Department of West China Hospital of Sichuan University. The patient’s relevant medical history was recorded, including gender, age of initial onset, onset eye, the interval between onset and the first visit to our hospital, extraocular muscle condition, smoking status, CAS, NOSPECS, thyroid function, symptoms and signs, and the condition of the affected extraocular muscle. We found that IMRT combined with periorbital triamcinolone acetonide injection has a good therapeutic effect on TED patients with low CAS but significant active extraocular muscles, and there is a significant improvement at 3 months after treatment. Thyroid function and CAS can affect the curative effect of IMRT. Patients with hyperthyroidism had worse effect than those with normal thyroid function. The higher the CAS, the better the effect.

**Supplementary Information:**

The online version contains supplementary material available at 10.1038/s41598-025-88142-w.

## Introduction

Thyroid eye disease (TED) is an autoimmune disease linked to abnormal thyroid function and is the most common orbital disease observed in clinical settings^1,2^. Clinical signs and symptoms include tearing, eye pain, eyelid retraction, upper eyelid lag, exophthalmos, and diplopia. In severe cases, corneal and optic nerve damage can significantly impact patients’ vision and quality of life^3^. According to the 2021 European Group on Graves’ Orbitopathy (EUGOGO) clinical practice guidelines for the medical management of Graves’ orbitopathy, glucocorticoid pulse therapy is the first-line treatment for moderate-to-severe active TED, while orbital radiotherapy serves as the second-line treatment^4^. However, clinical observations indicate that many Chinese TED patients with significant active extraocular muscles but low CAS experience less effectiveness from glucocorticoid pulse therapy than expected, whereas IMRT may lead to a better prognosis. This study aims to assess the curative effect evaluation of IMRT combined with periorbital triamcinolone acetonide injection for treating TED patients with significant active extraocular muscles but low CAS, offering insights for the clinical management of these patients.

## Materials and methods

### Population enrollment and evaluation

The retrospective observational study was conducted. A total of 156 eligible patients were selected from the TED patient database of the Ophthalmology Department of West China Hospital of Sichuan University. The patient’s relevant medical history was recorded, including gender, age of initial onset, onset eye, the interval between onset and the first visit to our hospital, extraocular muscle condition, smoking status, CAS, NOSPECS (a grading system of Graves’ orbitopathy[5]), thyroid function, and symptoms and signs. The maximum area of the coronal position of the affected extraocular muscle and the degree of enhanced MRI of T1-weighted and T2-weighted images were measured using the built-in software of YINGLIAN image system (The principle is to measure the degree of strengthening of the affected muscle in the enhanced MRI, and then calculate the ratio with the degree of strengthening of the ipsilateral temporal muscle as a reference). The degree of enhanced MRI of T1-weighted images of less than 30% is considered mild inflammation of the extraocular muscle, between 30% and 60% is moderate inflammation, and greater than 60% is severe inflammation. The degree of enhanced MRI of T2-weighted images of less than 30% is considered mild edema of the extraocular muscle, between 30% and 60% is moderate edema, and greater than 60% is severe edema. Follow-up examinations were conducted at one and three months after treatment. An enhanced MRI was performed three months after treatment.

The inclusion criteria for patients: (1) Patients who met the diagnostic criteria of TED were combined with medical history, clinical features and imaging examination^5^; (2) The patient’s CAS was <3. (3) The mean score of extraocular muscle inflammation was ≥ 1.5 (Mild inflammation of the extraocular muscle is scored as 1, moderate inflammation as 2, and severe inflammation as 3. The mean inflammation score of the extraocular muscle is calculated as the total inflammation score of all involved muscles divided by the number of involved muscles); (4) Mean of the maximum area of the coronal position of all the affected extraocular muscle was ≥ 45.0 mm^2^^6^; (5) The patient has complete medical records and enhanced MRI images; (6) The patient received a full course of IMRT in our hospital. The exclusion criteria: (1) The patient’s medical records were missing seriously. (2) The patient lost to follow-up or died because of other diseases. (3) The patient received IMRT for TED or decompressive surgery before enrollment; (4) The patient treated with other radiotherapy techniques.

This study was approved by the Biomedical Ethics Sub-Committee of the West China Hospital of Sichuan University. Requirement of informed consent was waived by the Biomedical Ethics Sub-Committee of the West China Hospital of Sichuan University, because it was a retrospective study. All methods were performed in accordance with the relevant guidelines and regulations.

## Specific program of therapy

All patients accepted the combination therapy, which involved IMRT and three periorbital triamcinolone acetonide (TA) injections (At the beginning of IMRT treatment, the first dose is administered, followed by every other week for a total of three doses).

The steps for periorbital TA injection were as follows: patients were positioned horizontally during the injection process. After disinfecting the periocular skin with povidone-iodine solution, a 26-gauge disposable needle was used by the same physician to inject a total of 40 mg of TA (Kunming Jida Pharmaceutical Co., LTD) into each orbit. The distribution of the 40 mg dose of TA varied based on the affected extraocular muscles. If only the upper eyelid with superior or medial rectus muscles was involved, 40 mg of TA was injected in the superior inner quadrant of the orbit. Similarly, when the inferior rectus muscle was the only one involved, 40 mg of TA was injected in the inferolateral orbit. In cases where the upper eyelid and multiple muscles, including the inferior rectus muscle, were involved, 20 mg of TA was injected in the superior inner and inferolateral quadrants of the orbit, respectively. Emergency situations such as high orbital pressure or arrhythmia served as stop signals for injection. Once the injection was complete, patients were instructed to press onto the injection site for 20 min.

In this study, reversely planned 7-field IMRT was employed as the OR treatment strategy. To begin, patients were immobilized using a customized thermoplastic cast. A CT scan with a slice thickness of 3 mm was performed for imaging acquisition and target contouring. The clinical target volume included the main bulk, origins, and insertions of extraocular muscles, as well as retro-orbital fat of the target eye. A planning target volume was created with a 2 mm concentric margin around the clinical target volume. The globe, lens, and optic nerve were outlined as organs-at-risk. Patients received a total dose of 20 Gy in 10 fractions over a two-week period^7^.

## Curative effect evaluation

The TED scoring system, developed from prior research, evaluates 13 symptoms and signs: eye pain, tearing, diplopia, and other symptoms (e.g., photophobia, foreign body sensation, blurred vision, dry eye), as well as upper eyelid lag, eyelid retraction, exophthalmos, soft tissue involvement, eye movement limitations, extraocular muscle inflammation, extraocular muscle edema, corneal involvement, and optic nerve involvement^8–10^. The TED score, which ranges from 0 to 31 points, represents the sum of individual item scores and is provided in Supplementary Table S1 online. Efficacy is primarily evaluated by calculating the efficacy ratio. The efficacy ratio is calculated as follows: X0 is the TED score plus the maximum area of the coronal position of the affected extraocular muscle before treatment. X1 is calculated by the same algorithm after treatment. The curative effect after treatment is calculated as (X0 − X1) / X0, and the result is expressed as a percentage. Efficacy is categorized into four levels based on the efficacy ratio: no improvement (ratio ≤ 0%), mild improvement (0% < ratio ≤ 10%), moderate improvement (10% < ratio ≤ 33%), and significant improvement (33% < ratio ≤ 100%). For single indicators, effectiveness is evaluated using the same formula and criteria. Patients with normal baseline values that remained unchanged after treatment are excluded from the analysis.

### Statistical analysis

The data of eyes of all research subjects were analyzed by the STATISTICAL ANALYSIS SYSTEM (version 9.4). For quantitative data, the mean ± standard deviation was used, and categorical variables were represented by the number of cases (constituent ratio). The Kolmogorov‒Smirnov test was used to test the normality of variables. A linear mixed model, generalized estimating equations (GEE) approach or logistic regression model were used to explore the influencing factors. *P* < 0.05 indicates a statistically significant difference.

## Results

### The basic characteristics of the research subjects before treatment

**Fig. 1:**
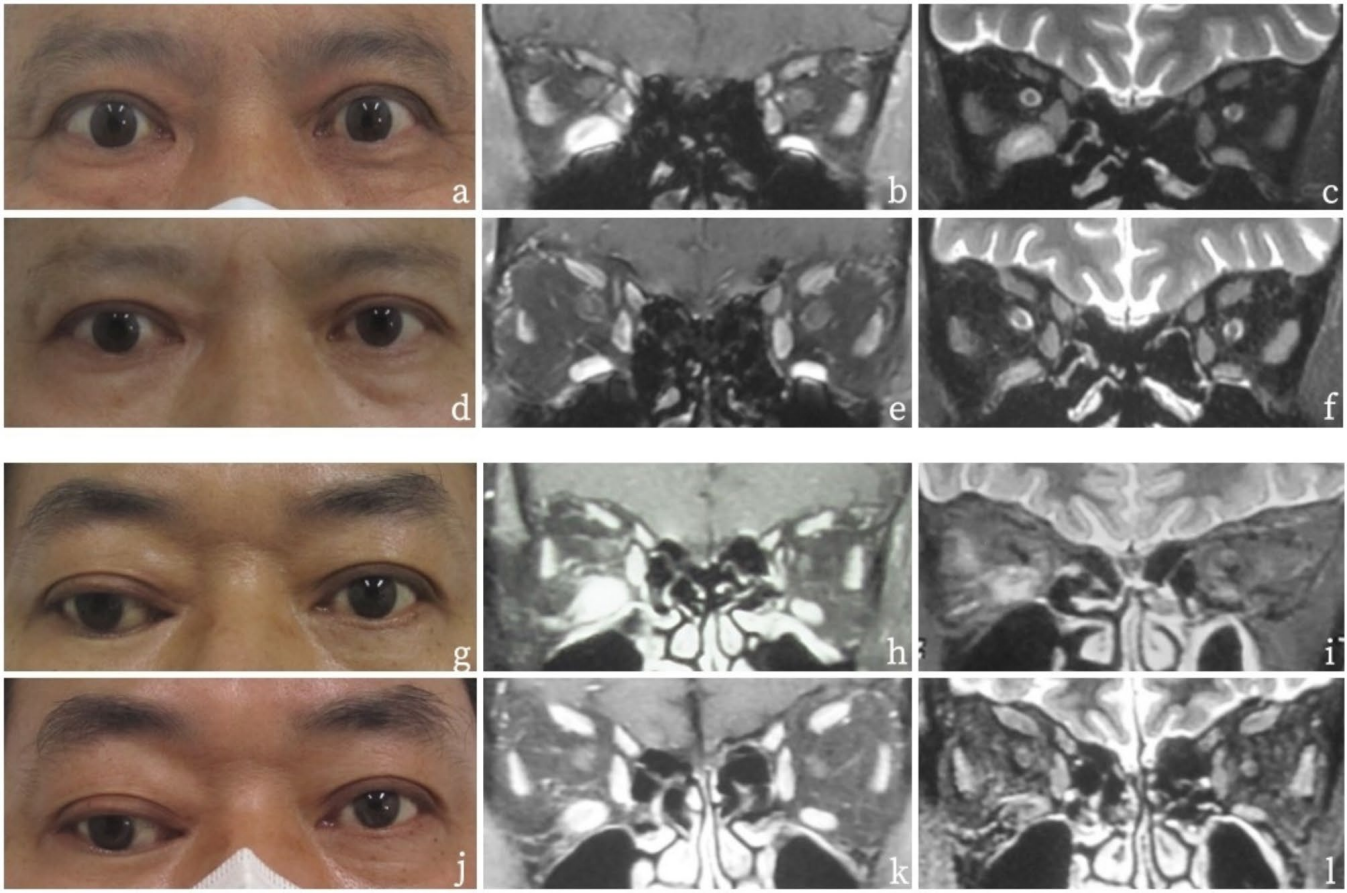
Legends, (**a**–**f**): A TED patient with no significant abnormality in ocular appearance, presenting only with limited eye movement. MRI revealed active extraocular muscles. (**a–c**): Images at the initial visit; (**d**–**f**): Images at three months post-IMRT. (**g**–**l**): A TED patient with pronounced restrictive strabismus. MRI showed active extraocular muscles. (**g**–**i**): Images at the initial visit; (j-l): Images at three months post-IMRT. (**a**, **d**, **g**, **j**): Ocular appearance; (**b**, **e**, **h**, **k**): T1-weighted orbital enhanced MRI images; (**c**, **f**, **i**, **l**): T2-weighted orbital enhanced MRI images.

A total of 156 patients were enrolled, including 108 females and 48 males (mean age: 47.66 ± 11.79 years, with an interval of 8.54 ± 11.74 months between onset and diagnosis). Monocular onset occurred in 34.6% of cases, while binocular onset accounted for 65.4%. Normal thyroid function was observed in 13.5% of patients, while 85.5% had abnormal thyroid function (83.3% with hyperthyroidism and 3.2% with hypothyroidism). Among the patients, 37.8% were smokers, while 62.2% had never smoked. Figure 1 shows the ocular appearance and orbital enhanced MRI images of a TED patient with active extraocular muscle but low CAS. Table 1 presents the baseline characteristics of the subjects before treatment. At the initial hospital visit, all patients presented with varying degrees of extraocular muscle thickening and inflammation, and 60.9% showed signs of extraocular muscle edema. Among TED patients with active extraocular muscle but low CAS before treatment, the frequency of extraocular muscle involvement is ranked from high to low as the inferior rectus muscle, superior rectus muscle, medial rectus muscle, and external rectus muscle. Table 2 shows the specific situation of the patient involved extraocular muscle.

**Table 1:** The basic characteristics of patients before and after treatment.

	Before treatment	After treatment
CAS		
0	24 (15.4)	50 (32.1)
1	86 (55.1)	86 (55.1)
2	46 (29.5)	17 (11.0)
3	0 (0.0)	1 (0.6)
4	0 (0.0)	1 (0.6)
5	0 (0.0)	1 (0.6)
NOSPECS		
0	0 (0.0)	1 (0.6)
1	0 (0.0)	1 (0.6)
4	156 (100.0)	153 (98.2)
5	0 (0.0)	1 (0.6)
Eye pain		
Negative	127 (81.4)	130 (83.3)
Positive	29 (18.6)	26 (16.7)
Tearing		
Negative	105 (67.3)	122 (78.2)
Positive	51 (32.7)	34 (21.8)
Diplopia		
Negative	41 (26.3)	49 (31.4)
Intermittent occurrence, often appearing when tired or walking	78 (50.0)	97 (62.2)
Non persistent presence, often appearing in the secondary position of eye or blinking	30 (19.2)	9 (5.8)
Persistent existence, often occurring in primary position of eye, viewing near objects, or reading	7 (4.5)	1 (0.6)
Other related symptoms *		
Negative	98 (62.8)	128 (82.1)
Positive	58 (37.2)	28 (17.9)
Upper eyelid lag		
Negative	28 (17.9)	49 (31.4)
Positive	128 (82.1)	107 (68.6)
Eyelid retraction		
Negative	61 (39.1)	82 (52.6)
The upper eyelid retraction was 1–2 mm	63 (40.4)	64 (41.0)
The upper eyelid retraction was 3–4 mm	31 (19.9)	10 (6.4)
The upper eyelid retraction was more than 5 mm	1 (0.6)	0 (0.0)
Soft tissue involvement		
Negative	28 (17.9)	64 (41.0)
Swelling of eyelids, lacrimal glands and lacrimal mounds; Congestive edema of conjunctiva	126 (80.8)	91 (58.3)
Obvious edema of conjunctival bulbar; Lagophthalmus	2 (1.3)	1 (0.6)
Exophthalmos		
Negative	153 (98.1)	155 (99.4)
Exophthalmometric measurement: >14 mm and ≤ 17 mm	3 (1.9)	1 (0.6)
Eye movement		
Negative	2 (1.3)	12 (7.7)
Limited eye movement, evident in extreme rotation in one or more directions	100 (64.1)	119 (76.3)
Significant limited eye movement	50 (32.1)	22 (14.1)
The position of the eyeballs is fixed	4 (2.6)	3 (1.9)
Extraocular muscle inflammation		
Negative	0 (0.0)	39 (25.0)
Positive	156 (100.0)	117 (75.0)
Extraocular muscle edema		
Negative	61 (39.1)	121 (77.6)
Positive	95 (60.9)	35 (22.4)
Cornea involvement		
Negative	156 (100.0)	155 (99.4)
Punctate defect of corneal epithelium	0 (0.0)	1 (0.6)
Optic nerve involvement		
Negative	49 (31.4)	60 (38.5)
The optic disc is ischemic and gray; Visual field defect; 0.3 ≤ Visual acuity <1.0	95 (60.9)	90 (57.7)
0.1 ≤ Visual acuity <0.3	6 (3.8)	5 (3.2)
No light perception; Visual acuity <0.1	6 (3.8)	1 (0.6)

* Other related symptoms include photophobia, foreign body sensation, blurred vision, dry eye.

**Table 2:** The specific situation of the patient involved extraocular muscle before treatment.

Parameter	Inflammation *N*(%)	Edema *N*(%)
Inferior rectus muscle of left eye		
0	54 (34.6)	101 (64.8)
1	13 (8.3)	25 (16.0)
2	33 (21.2)	24 (15.4)
3	56 (35.9)	6 (3.8)
Medial rectus muscle of left eye		
0	108 (69.2)	141 (90.4)
1	17 (10.9)	6 (3.8)
2	24 (15.4)	8 (5.2)
3	7 (4.5)	1 (0.6)
Superior rectus muscle of left eye		
0	101 (64.8)	140 (89.7)
1	8 (5.1)	6 (3.8)
2	23 (14.7)	10 (6.5)
3	24 (15.4)	0 (0.0)
Lateral rectus muscle of left eye		
0	136 (87.1)	154 (98.8)
1	4 (2.6)	1 (0.6)
2	14 (9.0)	1 (0.6)
3	2 (1.3)	0 (0.0)
Inferior rectus muscle of right eye		
0	46 (29.5)	103 (66.0)
1	12 (7.7)	21 (13.5)
2	45 (28.8)	29 (18.6)
3	53 (34.0)	3 (1.9)
Medial rectus muscle of right eye		
0	118 (75.7)	147 (94.2)
1	10 (6.4)	4 (2.6)
2	20 (12.8)	5 (3.2)
3	8 (5.1)	0 (0.0)
Superior rectus muscle of right eye		
0	103 (66.1)	135 (86.5)
1	7 (4.5)	11 (7.1)
2	23 (14.7)	9 (5.8)
3	23 (14.7)	1 (0.6)
Lateral rectus muscle of right eye		
0	137 (87.8)	150 (96.2)
1	8 (5.1)	4 (2.6)
2	7 (4.5)	1 (0.6)
3	4 (2.6)	1 (0.6)

## The basic characteristics of the research subjects after treatment

Table 1 shows the basic characteristics of patients after treatment and Table 3 shows the specific situation of the patient involved extraocular muscle after treatment.

**Table 3:** The specific situation of the patient involved extraocular muscle after treatment.

Parameter	Inflammation *N*(%)	Edema *N*(%)
Inferior rectus muscle of left eye		
0	89 (57.0)	137 (87.8)
1	51 (32.7)	17 (10.9)
2	16 (10.3)	2 (1.3)
3	0 (0.0)	0 (0.0)
Medial rectus muscle of left eye		
0	133 (85.3)	150 (96.2)
1	17 (10.9)	3 (1.9)
2	6 (3.8)	3 (1.9)
3	0 (0.0)	0 (0.0)
Superior rectus muscle of left eye		
0	126 (80.8)	152 (97.5)
1	22 (14.1)	3 (1.9)
2	8 (5.1)	1 (0.6)
3	0 (0.0)	0 (0.0)
Lateral rectus muscle of left eye		
0	146 (93.6)	154 (98.8)
1	8 (5.1)	1 (0.6)
2	2 (1.3)	1 (0.6)
3	0 (0.0)	0 (0.0)
Inferior rectus muscle of right eye		
0	85 (54.5)	135 (86.5)
1	57 (36.5)	20 (12.9)
2	14 (9.0)	1 (0.6)
3	0 (0.0)	0 (0.0)
Medial rectus muscle of right eye		
0	137 (87.8)	151 (96.8)
1	16 (10.3)	3 (1.9)
2	3 (1.9)	2 (1.3)
3	0 (0.0)	0 (0.0)
Superior rectus muscle of right eye		
0	118 (75.7)	151 (96.8)
1	25 (16.0)	5 (3.2)
2	12 (7.7)	0 (0.0)
3	1 (0.6)	0 (0.0)
Lateral rectus muscle of right eye		
0	149 (95.5)	155 (99.4)
1	5 (3.2)	0 (0.0)
2	2 (1.3)	1 (0.6)
3	0 (0.0)	0 (0.0)

## The curative effect evaluation

**Fig. 2:**
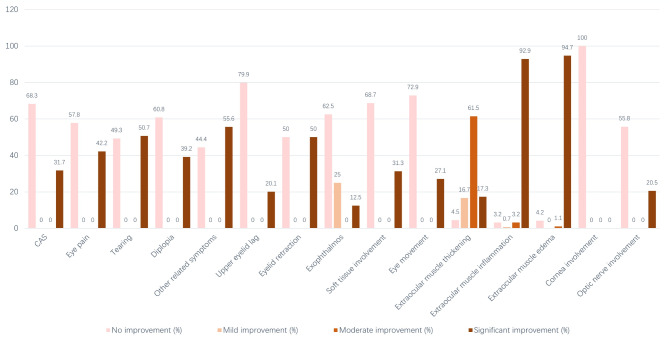
Legends, The curative effect evaluation results of each single indicator after treatment.

Based on curative effect evaluation of TED scoring, 1.3% of patients showed no improvement, while 98.7% demonstrated improvement: 3.8% mild, 18.6% moderate, and 76.3% significant. Figure 2 shows the curative effect evaluation results of each single indicator.

### Factors affecting the curative effect evaluation of IMRT combined with periorbital triamcinolone acetonide injection based on TED scoring

Univariate generalized linear regression analysis was performed, and when *P*<0.10, it was considered that the indicators had a statistically significant impact on the results. Take meaningful indicators from the univariate analysis results. Then, conduct a multivariate generalized linear regression analysis. When *P*<0.05, the risk factors were considered to have a statistically significant impact on the results. In conclusion, patients with hyperthyroidism had worse outcomes than those with normal thyroid function (*P* < 0.05). A higher CAS was associated with better outcomes (*P* < 0.01) (Table 4).

**Table 4:** Risk factors affecting the curative effect evaluation of IMRT combined with periorbital triamcinolone acetonide injection based on TED scoring.

Parameter	Estimate	StdErr	Wald χ²	*P* value
Thyroid function				
Hyperthyroidism	− 1.136	0.525	4.690	0.030
Hypothyroidism	− 0.042	1.120	0.001	0.970
Normal	–	–	–	–
CAS	0.579	0.163	12.568	< 0.001

## Discussion

In this study, we analyzed 156 TED patients with low CAS but significant active extraocular muscles. The mean age was approximately 47 years, with a higher proportion of females. Among patients, 65.4% had binocular onset, and 83.3% had hyperthyroidism.

It has been reported that 80–90% of TED patients are associated with thyroid dysfunction^11^. In this study, 83.3% of TED patients had hyperthyroidism. Thyroid function was closely linked to the IMRT curative effect in TED patients with low CAS but significant active extraocular muscles. We found that patients with hyperthyroidism had poorer outcomes compared to those with normal thyroid function.

All patients had different degrees of extraocular muscle thickening and inflammation before the treatment, and 60.9% of patients had different degrees of extraocular muscle edema. The frequency of extraocular muscle involvement reported in various literatures at home and abroad is different, but the inferior rectus muscle involvement is the most common. In our study, whether it was extraocular muscle thickness, extraocular muscle inflammation or extraocular muscle edema, the frequency of extraocular muscle involvement is ranked from high to low as the inferior rectus muscle, superior rectus muscle, medial rectus muscle, and external rectus muscle. This finding aligns with other reports showing that inferior rectus muscle involvement is the most common^12–15^.

This study found that 98.7% of patients had varying degrees of improvement, a much higher rate than previous studies. This may be because TED patients in other studies had both high CAS and significant active extraocular muscles, while our patients had lower activity levels compared with them^16^. All indicators except corneal involvement showed improvement after treatment. Improvement rates were highest for extraocular muscle inflammation (96.8%), followed by edema (95.8%) and thickening (95.5%). The lowest rates were for corneal involvement (0.0%), upper eyelid lag (20.1%), and extraocular edema (27.1%). This study found that for TED patients with low CAS but significant active extraocular muscles, the higher the CAS, the better the therapeutic effect of treatment. The efficacy of patients with hyperthyroidism is worse than that of patients with normal thyroid function. This is similar to previous research^7^. In previous studies, current smoking and symptom duration longer than 18 months were identified as independent predictive factors for non-response of TED to retro-orbital IMRT^17^. However, smoking and long symptom duration were not found to affect the therapeutic effect in our study. This may be because patients in our study had a relatively short disease duration, mostly within 12 months, and we used a different outcome evaluation system.

These results suggest that IMRT combined with periorbital triamcinolone acetonide injection has a good therapeutic effect on TED patients with low CAS but significant active extraocular muscles.

At the same time, there are certain limitations to our research. The three-month follow-up period in this study is indeed relatively short, and the need for longer-term data to fully assess the sustained efficacy of the treatment. At this stage, the primary goal of our study is to evaluate the short-term therapeutic effects, and if clear benefits are observed, we plan to conduct further research to assess the long-term outcomes. Additionally, the absence of a control or comparison group is a limitation that makes it challenging to attribute the observed improvements solely to the intervention. We chose not to include a control group because excluding TED patients with similar conditions from treatment would have been ethically challenging. We recognize that this decision introduces some limitations, but it aligns with our priority of providing care to all eligible patients.

## Conclusion

IMRT combined with periorbital triamcinolone acetonide injection has a good therapeutic effect on TED patients with low CAS but significant active extraocular muscles, and there is a significant improvement at 3 months after treatment. Thyroid function and CAS can affect the curative effect of IMRT combined with periorbital triamcinolone acetonide injection. Patients with hyperthyroidism had worse curative effect than those with normal thyroid function. The higher the CAS, the better the curative effect.

## Electronic supplementary material

Below is the link to the electronic supplementary material.

Supplementary Material 1

## Data Availability

Data sets generated during the current study are available from the corresponding author on reasonable request.
