# Supplementary material for: A curative effect evaluation of intensity-modulated radiation therapy combined with periorbital triamcinolone acetonide injection in treating thyroid eye disease patients with active extraocular muscle but low CAS
Source: Sci Rep. 2025 Jan 25;15:3222. doi: 10.1038/s41598-025-88142-w (PMC11763082; doi:10.1038/s41598-025-88142-w)
Supplement: Supplementary file 1 — Supplementary Material 1 [file 41598_2025_88142_MOESM1_ESM.docx]

**Table 1. The TED scoring system**

|  | Score | Standard |
| --- | --- | --- |
| Eye pain | 0 | Negative |
|  | 1 | Positive |
| Tearing | 0 | Negative |
|  | 1 | Positive |
| Diplopia | 0 | Negative |
|  | 1 | Intermittent occurrence, often appearing when tired or walking |
|  | 2 | Non persistent presence, often appearing in the secondary position of eye or blinking |
|  | 3 | Persistent existence, often occurring in primary position of eye, viewing near objects, or reading |
| Other related symptoms * | 0 | Negative |
|  | 1 | Positive |
| Upper eyelid lag | 0 | Negative |
|  | 1 | Positive |
| Eyelid retraction | 0 | Negative |
|  | 1 | The upper eyelid retraction was 1-2mm |
|  | 2 | The upper eyelid retraction was 3-4mm |
|  | 3 | The upper eyelid retraction was more than 5mm |
| Soft tissue involvement | 0 | Negative |
|  | 1 | Swelling of eyelids, lacrimal glands and lacrimal mounds; Congestive edema of conjunctiva |
|  | 2 | Obvious edema of conjunctival bulbar; Lagophthalmus |
|  | 3 | Severe edema of the bulbar conjunctival protruding beyond the palpebral fissure |
| Exophthalmos | 0 | Negative |
|  | 1 | Exophthalmometric measurement：＞14mm and ≤17mm |
|  | 2 | Exophthalmometric measurement：＞17mm and ≤21mm |
|  | 3 | Exophthalmometric measurement：＞21mm |
| Eye movement | 0 | Negative |
|  | 1 | Limited eye movement, evident in extreme rotation in one or more directions |
|  | 2 | Significant limited eye movement |
|  | 3 | The position of the eyeballs is fixed |
| Extraocular muscle inflammation | 0 | Negative |
|  | 1 | The degree of enhanced MRI of T1-weighted images：＜30% |
|  | 2 | The degree of enhanced MRI of T1-weighted images：≥30% and ＜60% |
|  | 3 | The degree of enhanced MRI of T1-weighted images：≥60% |
| Extraocular muscle edema | 0 | Negative |
|  | 1 | The degree of enhanced MRI of T2-weighted images：＜30% |
|  | 2 | The degree of enhanced MRI of T2-weighted images：≥30% and ＜60% |
|  | 3 | The degree of enhanced MRI of T2-weighted images：≥60% |
| Cornea involvement | 0 | Negative |
|  | 1 | Punctate defect of corneal epithelium |
|  | 2 | Corneal ulcer |
|  | 3 | Corneal nebula; Corneal necrosis; Corneal perforation |
| Optic nerve involvement | 0 | Negative |
|  | 1 | The optic disc is ischemic and gray; Visual field defect; 0.3≤Visual acuity ＜1.0 |
|  | 2 | 0.1≤Visual acuity ＜0.3 |
|  | 3 | No light perception; Visual acuity ＜0.1 |

Note: * Other related symptoms include photophobia, foreign body sensation, blurred vision, dry eye.
